# The role of splicing events in the inflammatory response of atherosclerosis: molecular mechanisms and modulation

**DOI:** 10.3389/fimmu.2024.1507420

**Published:** 2024-12-17

**Authors:** Aolong Wang, Chengzhi Wang, Bihan Xuan, Yanqin Sun, Bin Li, Qifei Zhao, Rui Yu, Xinlu Wang, Mingjun Zhu, Jingjing Wei

**Affiliations:** ^1^ Heart Center, The First Affiliated Hospital of Henan University of Chinese Medicine, Zhengzhou, China; ^2^ First Clinical Medical College, Henan University of Chinese Medicine, Zhengzhou, China; ^3^ Second Clinical Medical College, Henan University of Chinese Medicine, Zhengzhou, China; ^4^ College of Integrated Traditional Chinese and Western Medicine, Fujian University of Traditional Chinese Medicine, Fuzhou, China; ^5^ Henan Evidence-Based Medicine Center of Chinese Medicine, The First Affiliated Hospital of Henan University of Chinese Medicine, Zhengzhou, China

**Keywords:** atherosclerosis, splicing events, inflammatory response, molecular mechanisms, biomarker

## Abstract

Atherosclerosis is a chronic inflammatory disease characterized by persistent inflammatory responses throughout all stages of its progression. Modulating these inflammatory responses is a promising avenue for the development of cardiovascular disease therapies. Splicing events modulate gene expression and diversify protein functionality, exerting pivotal roles in the inflammatory mechanisms underlying atherosclerosis. These insights may provide novel opportunities for developing anti-inflammatory therapies for this disease. This article systematically discusses the diverse splice variants and how splicing events impact the inflammatory response in atherosclerosis via endothelial cells, macrophages, and vascular smooth muscle cells, highlighting their underlying molecular mechanisms and implications. Furthermore, this study summarizes clinical evidence supporting splicing-related molecules as diagnostic biomarkers and therapeutic targets in atherosclerosis. Lastly, we outline the current challenges and future research directions concerning splicing events and inflammatory responses in atherosclerosis. This offers a novel perspective and evidence for formulating new therapeutic strategies aimed at lowering the risk of atherosclerosis.

## 1 Introduction

Atherosclerosis is a chronic inflammatory disease that serves as both a leading cause and the pathological basis of cardiovascular diseases (1). Approximately 500 million people worldwide currently suffer from cardiovascular diseases, which account for about one-third of total global deaths (2, 3). Despite significant reductions in cardiovascular mortality achieved through existing atherosclerosis treatment strategies (4), the persistently high incidence underscores the necessity for novel preventive and management approaches, marking a critical focus for future research (5). The pathological characteristics of atherosclerosis include vascular endothelial damage, lipid accumulation, and the formation of oxidized low-density lipoprotein (oxLDL) (6). Endothelial cells (ECs), monocytes/macrophages, vascular smooth muscle cells (VSMCs), dendritic cells, B cells, and T cells all participate in the atherosclerotic process (7, 8). Inflammatory responses act as key drivers of atherosclerosis progression, exacerbating the risk of residual cardiovascular complications. Anti-inflammatory strategies targeting atherosclerosis hold significant potential for future therapeutic development (9, 10).

RNA splicing is an essential mechanism for the formation of both coding mRNA and certain non-coding RNAs (11). Splicing events encompass constitutive splicing, alternative splicing, and trans-splicing, with approximately 95% of human genes linked to alternative splicing (12). The accuracy of RNA splicing is critical for maintaining normal gene function and cellular homeostasis (13). Recent research has indicated that splicing events play a broad role in asthma, chronic obstructive pulmonary disease, and chronic inflammatory diseases such as atherosclerosis (14, 15). Specifically, in atherosclerosis, alternative splicing regulates the activation, adhesion, and subsequent differentiation of monocytes into macrophages (16). Additionally, abnormal splicing events are closely associated with the inflammatory responses involving endothelial cells and vascular smooth muscle cells, contributing significantly to the progression of atherosclerosis. Inflammatory responses are interwoven throughout the pathological process of atherosclerosis, emerging evidence underscores the pivotal role of splicing-mediated inflammation in driving disease progression (**Table 1**) (17, 18). Investigating the molecular mechanisms underlying splicing events in atherosclerosis-related inflammatory responses is highly significant. We systematically analyzed the relationship between splicing events and inflammatory responses in atherosclerosis, aiming to highlight their potential as novel diagnostic biomarkers and therapeutic targets.

**Table 1:** The role of splicing events in the inflammatory response of atherosclerosis.

Regulatory factor	Experimental model	Splicing events	Molecular mechanism	References
**Ptbp1**	Mice	Alternative splicing associated with the TNF/NF-κB signaling pathway	Mediating nuclear localization and transcriptional activity of the RelA/p65 subunit in the NF-κB signaling pathway	(17)
**FUS, LncRNA *XXYLT1-AS2***	HUVECs	Apoptosis and proliferation are involved in atherosclerosis	Regulation of monocyte adhesion and levels of VCAM-1 and MCP-1 through the NF-κB pathway	(47, 48)
***XBP-1*, microRNA-512-3p**	HUVECs, Mice	The two isomers of *XBP-1* are XBP-1S and XBP-1U	Regulation of XBP-1S/XBP-1U ratio enhances vascular endothelial cell viability, inhibits apoptosis, and attenuates autophagy and endoplasmic reticulum stress	(50)
**lncRNA ANRIL, miR-181b**	HUVECs, Rats	Affects alternative splicing of genes involved in inflammatory response	Influencing the NF-κB signaling pathway by regulating miR-181b and regulating the release of inflammatory factors such as IL-6, IL-8, TNF-α, ICAM-1, and VCAM-1	(54)
**ANRIL, *NR_003529*, *DQ485454***	Human coronary artery tissue samples, HCAECs, HUVECs	Different transcripts of ANRIL *NR_003529* and *DQ485454*	Regulation of adhesion between monocytes and ECs	(56)
***SRSF10*, lncR-GAS5**	HUVECs	lncR-GAS5 regulates the expression of splicing factor *SRSF10*	Decreased LC3II/LC3I protein ratio, elevated P62 levels, and inhibited endothelial cell autophagy	(57)
**circ_0003645**	HUVECs		Attenuating the inflammatory response of the NF-κB pathway in HUVEC induced by oxLDL, such as the expression levels of IL-6, TNF-α, and NF-κB-related inflammatory proteins	(60)
**CircMETTL14 (11**)**S**	Mice		Modulation of endothelial cell inflammatory responses through the METTL14/CXCR4 axis	(61)
**CAPN6**	Mice, Bone Marrow Mononuclear Cell	Impact on the CWC22/EJC post-transcriptional splicing system	Modulation of inflammatory macrophage drinking capacity	(64)
***FOXP3*, *FOXP3fl*, *FOXP3Δ2***	CD4+ T cell, Tregs	Selective splicing of the FOXP3 gene	Regulation of immune function and inflammatory responses associated with Treg cells	(65)
**EDA^+^-FN**	Mice	EDA+-FN is produced by variable splicing from the fibronectin gene (*FN1*)	Activates TLR4 signaling and promotes onocyte/macrophage aggregation, enhances NF-κB p65 expression	(67, 68)
**NEAT1**	Mouse macrophages, THP-1 cells	NEAT1_2, the splice isoform of NEAT1, binds to RNA-binding proteins (PSPC1, NONO, and SFPQ) to form Paraspeckle	Involvement in ox-LDL-induced macrophage inflammation and oxidative stress in atherosclerosis; Influence on p65 protein phosphorylation regulates the production of inflammatory factor TNF-α	(72, 73)
**Elavl1**	Mice	Alternative splicing of adaptive immune-related genes	Regulation of T cell numbers and immunomodulatory proteins	(75, 76)
**Quaking**	Human carotid artery tissue, mice, THP-1 cells	Involved in pre-mRNA splicing and gene expression	Modulation of monocyte differentiation into pro-atherosclerotic macrophages	(16)
**Versican V3**	ASMCs		Induces smooth muscle cells to differentiate and exhibit anti-inflammatory properties	(82)
**MBNL1**	Human artery samples	Regulation of RNA splicing of *Abi1* gene	Activation of vascular smooth muscle cell-derived macrophage-like cell transdifferentiation through the Rac1-NOX1-ROS-KLF4 signaling pathway	(83)

Ptbp1, factor polypyrimidine tract-binding protein 1; TNF-α, Tumor necrosis factor-alpha; NF-κB, Nuclear factor kappa-light-chain-enhancer of activated B cells; FUS, Fused in sarcoma; HUVECs, human umbilical vein endothelial cells; VCAM-1, Vascular Cell Adhesion Molecule 1; MCP-1, monocyte chemoattractant protein-1; NF-κB, Nuclear factor kappa-light-chain-enhancer of activated B cells; XBP-1, X-Box binding protein 1; ANRIL, Antisense Non-Coding RNA in the INK4 Locus; IL-6, Interleukin-6; IL-8, Interleukin-8; TNF-α, Tumor necrosis factor-alpha; ICAM-1, Vascular Cell Adhesion Molecule 1;HCAECs, Human Coronary Artery Endothelial Cells; ECs, Endothelial Cells; lncR-GAS5, long non-coding RNA growth arrest-specific 5; SRSF10, Serine/Arginine-Rich Splicing Factor 10; METTL14, Methyltransferase-like 14; CXCR4, C-X-C chemokine receptor type 4; CAPN6, Calcium-activated neutral protease 6; FOXP3, Forkhead box P3; EDA+-FN, Extra Domain A Positive Fibronectin; FN, Fibronectin; TLR4, Toll-like receptor 4; NEAT1, Nuclear paraspeckle assembly transcript 1; Elavl1, Embryonic Lethal Abnormal Vision-Like 1; ASMCs, Airway Smooth Muscle Cells; MBNL1, Muscleblind-like splicing regulator 1; Abi1, Abl interactor 1.

Bold text indicates regulatory factors that play a pivotal role in modulating splicing events and inflammatory responses in atherosclerosis.

## 2 Pathological mechanisms and inflammatory responses in atherosclerosis

**Figure 1:**
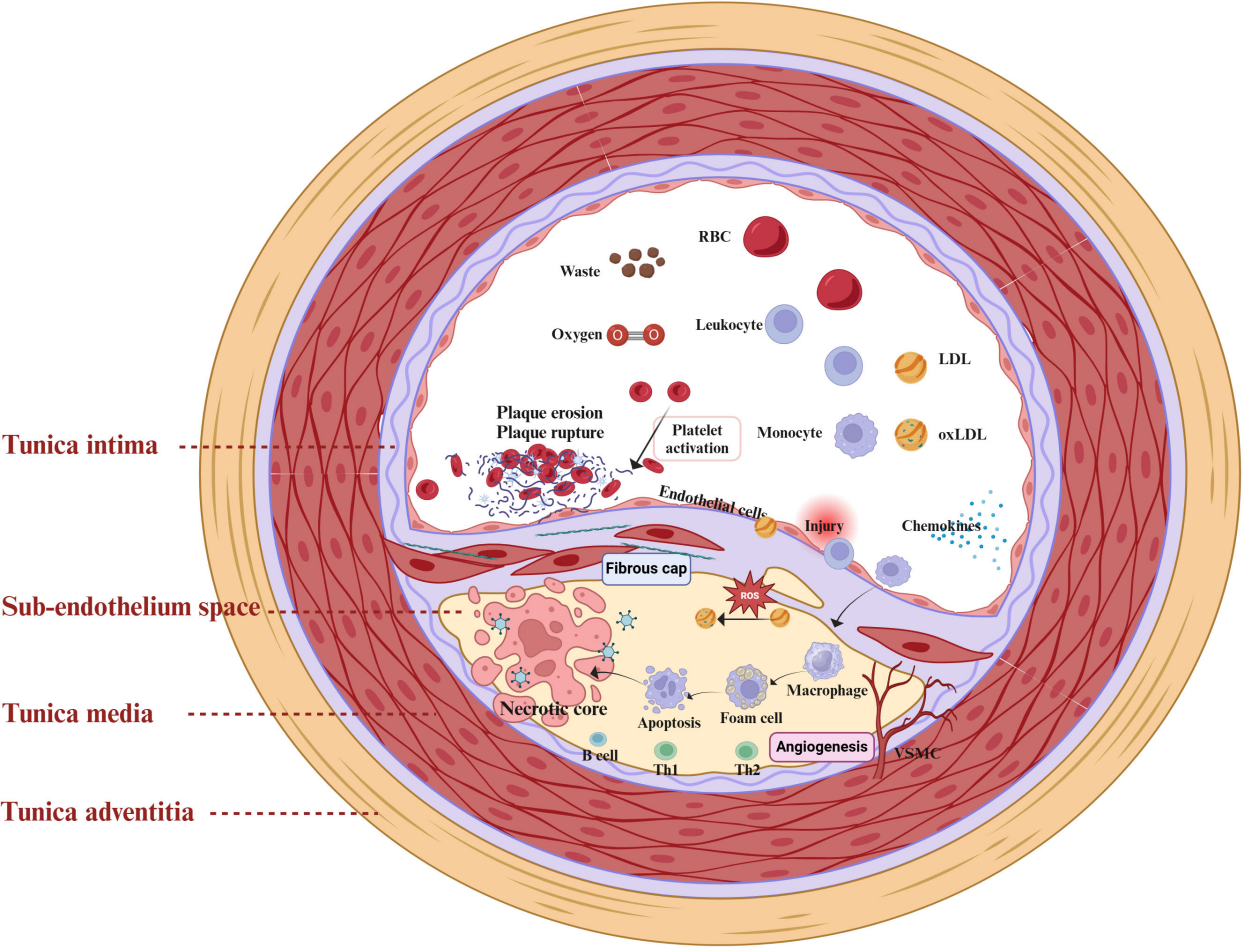
Mechanisms of atherosclerosis and the inflammatory response. The intricate interplay between diverse cellular populations and cytokines within the vascular microenvironment orchestrates the progression of atherosclerosis. The primary pathological mechanisms include Endothelial Cells (ECs) injury, dysregulated lipid metabolism and accumulation, macrophage recruitment, and proliferation and migration of Vascular Smooth Muscle Cells (VSMCs). Upon endothelial cell injury, the inflammatory response is activated, allowing low-density lipoprotein (LDL) to infiltrate the vascular intima where it accumulates and undergoes oxidation by reactive oxygen species (ROS), forming pro-inflammatory oxidized low-density lipoproteins (ox-LDL). Concurrently, immune-inflammatory cells, including T-lymphocytes, neutrophils, and monocytes, are continuously recruited and infiltrate emerging atheromatous plaques. Macrophages engulf ox-LDL, transforming into foam cells that contribute to the formation of a necrotic lipid core.

Atherosclerosis is a chronic inflammatory cardiovascular disease driven by multiple factors (19). The primary pathological mechanisms include ECs injury, dysregulated lipid metabolism and accumulation, macrophage recruitment, and proliferation and migration of VSMCs (**Figure 1**) (20). ECs injury and dysfunction are recognized as the initial stages of atherosclerosis (21). Atherosclerosis risk factors, including hypertension, hyperlipidemia, and hyperglycemia, contribute to endothelial damage. Following endothelial dysfunction, LDL infiltrates and accumulates within the vascular intima, where it undergoes oxidation by reactive oxygen species to form pro-inflammatory ox-LDL (22). The interaction between ox-LDL and proteoglycans exacerbates endothelial dysfunction, further promoting monocyte adhesion, accumulation, and differentiation into macrophages (23, 24). Macrophages engulf ox-LDL, resulting in the formation of foam cells. Activated immune inflammatory cells, such as T lymphocytes, neutrophils, and monocytes, persistently infiltrate atherosclerotic plaques. They release pro-inflammatory cytokines, including Tumor necrosis factor-alpha (TNF-α), Interleukin-1β(IL-1β), and Interleukin-6(IL-6) (25). Ultimately, the accumulation of foam cells impairs macrophage efferocytosis and triggers cell death (via apoptosis or necrosis), leading to the formation of a necrotic lipid core (26).

During the progression of atherosclerosis, endothelial cell injury and dysfunction, leukocyte recruitment, and foam cell formation activate inflammatory signaling pathways and release inflammatory factors, thereby promoting the formation and development of atherosclerotic plaques and increasing plaque instability (27). Endothelial dysfunction and inflammatory responses are tightly interconnected in atherosclerosis, where injured endothelial cells promote tissue repair by expressing inflammatory factors such as monocyte chemoattractant protein-1 (MCP-1), Interleukin-8(IL-8), selectins, chemokines, and adhesion molecules such as Vascular Cell Adhesion Molecule 1 (VCAM-1) and Intercellular Adhesion Molecule 1 (ICAM-1). Meanwhile, macrophages, VSMCs, and lymphocytes participate in the inflammatory response associated with atherosclerosis (28, 29). Foam cells predominantly originate from the differentiation of M2 macrophages (8), and VSMCs can transdifferentiate into macrophage-like cells (30), exacerbating endothelial injury. In addition, lymphocytes, encompassing both B cells and T cells, play intricate roles in the pathogenesis of atherosclerosis (31). Subsets of B cells exhibit dual functions, being both protective and pathogenic during the progression of atherosclerotic disease (27). Meanwhile, autoreactive effector T cells are capable of recognizing self-antigens such as oxLDL (30), thereby triggering inflammatory responses. Overall, chronic inflammation and immune dysregulation significantly influence the progression of atherosclerosis. Elucidating the underlying pathological mechanisms will deepen our understanding of this disease and foster the development of novel therapeutic strategies.

## 3 Concise of the splicing events

Splicing events are critical post-transcriptional processes essential for the formation of mature mRNA, encompassing constitutive splicing, alternative splicing, and trans-splicing (11). The process of removing introns from pre-mRNA and retaining exons, thereby connecting them to form mature mRNA, is known as constitutive splicing and is regulated by spliceosomes (32). Constitutive splicing is essential for sustaining fundamental cellular functions. Alternative splicing regulates the selective removal of introns from mRNA precursors while retaining specific exons (33), serving as a crucial factor in gene expression and protein functional diversity in multicellular eukaryotes (34, 35). Additionally, alternative splicing can interact with non-coding RNAs, including long non-coding RNAs (lncRNAs), circular RNAs (circRNAs), and microRNAs (miRNAs). While non-coding RNAs do not directly encode proteins, they influence gene transcription by regulating splicing events (36, 37). Recent research has shown that various splicing events and non-coding RNAs can mediate inflammatory responses in atherosclerosis by regulating RNA splicing (38–40). Considering the significant role of splicing events in the inflammatory response in atherosclerosis, we further investigated the interplay between splicing events, non-coding RNAs, and inflammation in this condition.

## 4 splicing events in atherosclerosis-related inflammatory responses

### 4.1 Regulation of inflammatory responses by splicing events in ECs

ECs, as critical regulators of immune cell aggregation and local inflammation, represent promising therapeutic targets for managing inflammatory responses (41). Risk factors for atherosclerosis, including hypertension and hyperglycemia, stimulate endothelial cells (42), activating the IκB kinase (IKK) complex, resulting in the phosphorylation and degradation of IκB, thereby releasing Nuclear factor kappa-light-chain-enhancer of activated B cells (NF-κB) into the nucleus,subsequently triggering the expression of pro-inflammatory molecules such as ICAM-1, VCAM-1, and MCP-1, which exacerbates endothelial cell injury (43, 44). Emerging evidence indicates that splicing events play a role in the inflammatory response associated with endothelial dysfunction by regulating the NF-κB signaling pathway (45). Under conditions of low and disturbed blood flow, the splicing factor polypyrimidine tract-binding protein 1(Ptbp1) is crucial for ECs activation and influences several alternative splicing events related to the TNF/NF-κB signaling pathway (46), mediating the nuclear localization and transcriptional activity of the RelA/p65 subunit in the NF-κB pathway, thus affecting the expression of inflammatory factors (17). Fused in sarcoma (FUS), an RNA-binding protein, is implicated in alternative splicing related to apoptosis and proliferation in atherosclerosis (47). Additionally, LncRNA *XXYLT1-AS2* can upregulate FUS expression, inhibiting the proliferation and adhesion of human umbilical vein endothelial cells (HUVECs), and regulating monocyte adhesion as well as the levels of VCAM-1 and MCP-1 via the NF-κB pathway (48). Furthermore, X-Box binding protein 1 (XBP1), as a transcription factor, plays a crucial role in atherosclerosis by regulating the inflammatory response (49). Recent studies have indicated that downregulation of microRNA-512-3p modulates the ratio of XBP-1S/XBP-1U by targeting *XBP-1*, enhancing vascular EC viability, inhibiting apoptosis, and alleviating autophagy and endoplasmic reticulum stress (50), thereby mitigating inflammation and slowing the progression of atherosclerosis.

**Figure 2:**
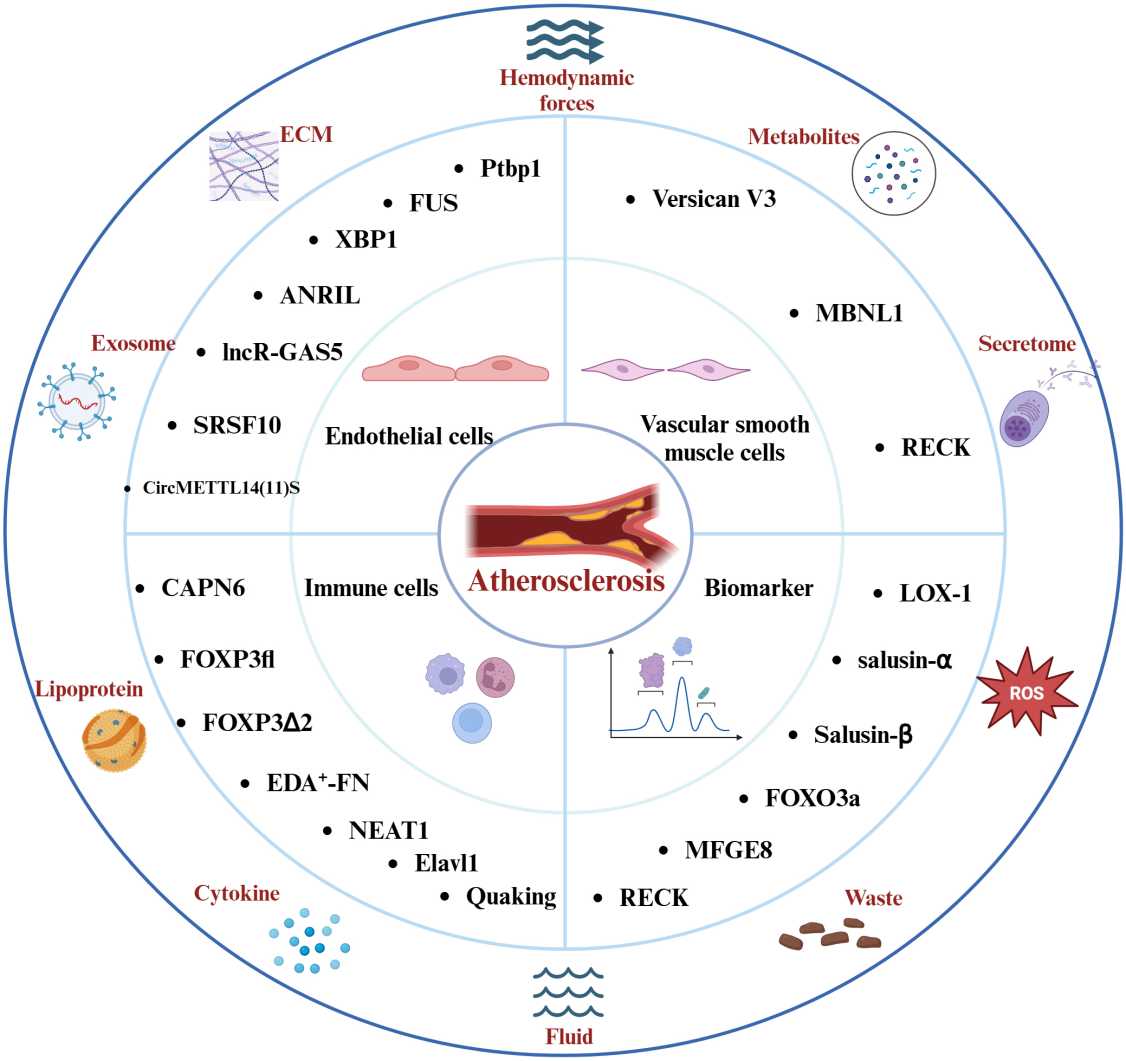
Factors Affecting the Atherosclerotic Inflammatory Response and the Molecules of Splicing Events Associated with the Atherosclerotic Inflammatory Response. Hemodynamics, lipoproteins, reactive oxygen species, and other elements play pivotal roles in the progression of this disease. The intricate interplay among endothelial cells, immune cells, vascular smooth muscle cells, and the inflammatory response constitutes the core pathological mechanism underlying atherosclerosis. Molecules involved in distinct splicing events modulate the inflammatory response in atherosclerosis and hold promise as potential diagnostic biomarkers and therapeutic targets for the disease.

Various non-coding RNAs also play a key role in the inflammatory response associated with atherosclerosis. The Antisense Non-Coding RNA in the INK4 Locus (ANRIL) is a multifunctional lncRNA that is closely associated with the inflammatory response (51–53). Further studies suggest that ANRIL modulates the NF-κB signaling pathway via miR-181b, subsequently affecting the release of inflammatory factors such as IL-6, IL-8, TNF-α, ICAM-1, and VCAM-1 (54). Inhibition of circANRIL expression can mitigate vascular endothelial injury and inflammatory responses in atherosclerotic rats models (55). Interestingly, alternative splicing generates distinct *ANRIL* transcripts, *NR_003529* and *DQ485454*, which exhibit opposing effects in coronary artery disease (CAD). Upregulation of *NR_003529* enhances the adhesion between monocytes and ECs, while downregulation of *DQ485454* has a comparable effect (56). Enhanced expression of long non-coding RNA growth arrest-specific 5 (lncR-GAS5) can reduce the LC3II/LC3I protein ratio and increase P62 levels, thereby inhibiting autophagic processes. This function of lncR-GAS5 is associated with the splicing factor Serine/Arginine-Rich Splicing Factor 10 (*SRSF10*) (57). Moreover, circRNAs participate in inflammatory responses related to endothelial cells (58, 59), circ_0003645 is another circRNA associated with atherosclerosis. Inhibition of circ_0003645 expression can reduce the inflammatory response in the NF-κB pathway induced by oxLDL in HUVECs, including the expression levels of IL-6, TNF-α, and NF-κB-related inflammatory proteins (60). Concurrently, CircMETTL14 (11) S exacerbates inflammation in ECs and promote the progression of atherosclerosis through the Methyltransferase-like 14 (METTL14)/C-X-C chemokine receptor type 4 (CXCR4) axis (61). In summary, endothelial cell injury plays a critical regulatory role in the inflammation associated with atherosclerosis. Splicing events and various non-coding RNAs participate in the progression of atherosclerotic disease by influencing endothelial cell function and inflammation (**Figure 2**). However, there seems to be a lack of extensive clinical studies to validate these observations. Conversely, the relationships between splicing events associated with the inflammatory response in atherosclerosis continue to pose challenges for our future research.

### 4.2 Regulation of inflammatory responses by splicing events in immune cells

Macrophages contribute to plaque formation in atherosclerosis by inducing chronic inflammation (62). Recent research has shown that specific splicing events influence the functions of macrophages and T cells, thereby modulating inflammatory response. Calcium-activated neutral protease 6 (CAPN6) is an atypical calpain enzyme (63). In atherosclerotic disease, *CAPN6* is specifically expressed in inflammatory macrophages. Notably, recent research has indicated that CAPN6 protein is induced in inflammatory conditions, resulting in dysregulation of the CWC22/EJC post-transcriptional splicing system, which further affects the phagocytic ability of inflammatory macrophages (64). Consequently, targeting CAPN6 or the associated CWC22/EJC system could represent an effective therapeutic strategy for atherosclerosis. Selective splicing of the Forkhead box P3 (*FOXP3*) gene mediates the effector functions of regulatory T cells and influences the stability of atherosclerotic plaques. *FOXP3fl* and *FOXP3Δ2* are distinct splice variants of *FOXP3*; *FOXP3Δ2* can influence Treg cell function, with low levels of FOXP3Δ2 associated with plaque instability. This may be connected to the role of *FOXP3Δ2* in Treg cell immune functions and inflammatory responses by regulating Glycoprotein A Repetitions Predominant (GARP) expression and its interaction with Transforming Growth Factor-beta (TGF-β) (65). In conclusion, selective splicing of *FOXP3* regulates the effector functions of regulatory T cells and is linked to the stability of atherosclerotic plaques. Fibronectin (FN) is a crucial extracellular matrix protein (18). Extra Domain A Positive Fibronectin (EDA+-FN) is a variant of FN generated by alternative splicing of *FN1* (66). EDA+-FN promotes the recruitment of monocytes/macrophages in atherosclerotic plaques through Toll-like receptor 4 (TLR4) signaling activation (67). Notably, EDA+-FN can further aggravate the inflammatory response in atherosclerosis by engaging integrin α5β1 and increasing NF-κB p65 expression (68).

Nuclear paraspeckle assembly transcript 1 (NEAT1) is a lncRNA involved in multiple pathological mechanisms in atherosclerosis, such as apoptosis, foam cell formation, and inflammation (69–71). NEAT1 interacts with miR-128 and is involved in ox-LDL-induced inflammation and oxidative stress response in macrophages during atherosclerosis (72). Notably, recent research has revealed that the splice isoform NEAT1_2 binds to RNA-binding proteins (PSPC1, NONO, and SFPQ) to form paraspeckles, which subsequently affect the phosphorylation of the p65 protein and regulate the production of the inflammatory factor TNF-α (73). The endothelial RNA-binding protein Elavl1 (HuR) modulates genes related to migration, inflammation, and adhesion, facilitating the recruitment and proliferation of T cells (74, 75). Additionally, HuR modulates pre-mRNA splicing and translation in endothelial cells (ECs), enhancing the expression of immune regulatory proteins, such as C1q and CD27, while reducing the population of CD8+ T cells (76). Moreover, the lncRNA *SUGCT-AS1* expression is significantly elevated in M1 macrophages, and it regulates the nuclear-cytoplasmic translocation of heterogeneous nuclear ribonucleoprotein U (hnRNPU) by binding, thereby influencing the alternative splicing of Mucosa-Associated Lymphoid Tissue Lymphoma Translocation Gene 1 *(MALT1)*. This process significantly impacts the NF-κB signaling pathway, as well as the expression and secretion of pro-inflammatory cytokines (77). Importantly, the RNA-binding protein Quaking plays a vital role in pre-mRNA splicing and gene expression, and it is regarded as a crucial transcriptional regulator in the differentiation of monocytes into pro-atherosclerotic macrophages (16). Extensive research has demonstrated that splicing events, along with related long non-coding RNAs and RBPs, play crucial roles in atherosclerosis (**Figure 2**). Future studies should focus on the regulation of genes responsible for the differentiation of monocytes into anti-inflammatory macrophages, offering a promising therapeutic approach to mitigating inflammation-driven atherosclerosis progression.

### 4.3 Regulation of inflammatory responses by splicing events in vsmcs

The phenotypic plasticity of VSMCs enables them to undergo transitions in response to pathological stimuli, such as pro-inflammatory factors and mechanical stretching (78), which is essential for the stability of atherosclerotic plaques (79). Versican, a multifunctional proteoglycan, is a significant high-molecular-weight glycoprotein that belongs to the glycosaminoglycan family (80). Among its splice variants, V3 is particularly notable for its ability to regulate adhesion plaque signaling pathways, modulating cell-extracellular matrix interactions (81). By influencing the expression of genes related to the complement system, chemokines, chemokine receptors, and transcription factors involved in inflammation, V3 promotes the differentiation of VSMCs and exerts anti-inflammatory effects (82). It is widely recognized that VSMCs can convert into macrophage-like cells during the process of atherosclerosis. Earlier research has shown that the dysregulation of Muscleblind-like splicing regulator 1 (MBNL1) can induce splicing events in the *Abi1* gene, leading to the production of a specific variant, *Abi1-Δe10*. This variant activates the transdifferentiation of VSMCs into macrophage-like cells by enhancing the Rac1-NOX1-ROS-KLF4 signaling pathway (83), potentially representing one of the mechanisms affecting inflammation in atherosclerosis.

Reversion-Inducing-Cysteine-Rich Protein with Kazal Motifs (RECK) is a membrane-anchored glycoprotein that modulates matrix metalloproteinases (MMPs) and is closely linked to inflammatory responses in cardiovascular diseases (84). Recent research indicates that OxLDL can enhance the proliferation and migration of VSMCs by inhibiting RECK expression via the NF-κB pathway (85). Conversely, overexpression of RECK suppresses IL-17A and MMP-13 expression, thereby diminishing SMC migration (86). Additionally, another study revealed that overexpression of RECK reduced the proliferation and migration of VSMCs induced by hypoxia and mediated by miR-195-5p, while inhibiting the protein expression of MMP-3, -9, and -13, as well as inflammatory responses (87). Notably, specific splice variants of RECK could act as potential biomarkers for coronary atherosclerotic disease (88). Furthermore, in a mouse model of atherosclerosis, circular RNA circEsyt2 interacts with PCBP1 to regulate its intracellular localization. Silencing circEsyt2 significantly enhances the interaction between the RNA splicing factor PCBP1 and U2AF65, leading to increased β-splicing of *p53*, which alters the expression of p53 target genes and inhibits VSMC proliferation (89). These findings underscore the intricate relationship between inflammatory responses and the regulation of VSMC proliferation, migration, and differentiation, which collectively influence plaque stability. Regulating splicing events to modulate the interactions between VSMCs and inflammatory responses seems to be a crucial strategy for maintaining plaque stability in AS moving forward (**Figure 2**).

## 5 The potential of splicing event-related molecules as biomarkers for the diagnosis and treatment of atherosclerosis

The risk for patients with atherosclerosis remains high, even with standardized treatment strategies, due to the crucial role of splicing events in the inflammatory response mechanisms of atherosclerosis, investigating splicing-related molecules as potential biomarkers for the diagnosis and therapeutic targets for atherosclerosis holds great promise (90). Lectin-like oxidized low-density lipoprotein receptor-1 (LOX-1) is closely related to atherosclerosis and is present in immune cells such as ECs, macrophages, and lymphocytes (91, 92). LOX-1 can activate inflammatory signaling pathways and molecules related to inflammation, including NF-κB and NLRP3 (93). Furthermore, it can upregulate cell adhesion molecules and MCP-1 via activation of the MAPK pathway, thereby facilitating the adhesion and infiltration of monocytes. Studies have shown that overexpression of the LOX-1 splicing variant Lectin-like Oxidized Low-Density Lipoprotein Receptor-1 Inhibitor (LOXIN) reduces ox-LDL-induced apoptosis, providing a protective effect (94, 95). In a 20-year prospective study, circulating LOX-1 was linked to the inflammatory marker High-sensitivity C-reactive protein (Hs-CRP), which elevated the risk of myocardial infarction, and this association remained significant even after adjusting for related risk factors (96). Numerous studies have identified LOX-1 as having distinct diagnostic and prognostic value in atherosclerosis (97–99).

Salusin-α and Salusin-β are bioactive peptides derived from the C-terminal alternative splicing of the torsin family member 2A (TOR2A) protein, exhibiting opposing roles in atherosclerosis (100). Previous research has demonstrated that salusin-β mediates the NF-κB signaling pathway and is involved in the inflammatory response of endothelial cells (101), enhancing the expression of inflammatory factors including VCAM-1, MCP-1, IL-6, IL-8, IL-1β, and NADPH oxidase 2 (102). Notably, a clinical trial revealed a significant difference in salusin-β levels between CAD patients and the control group, highlighting its potential as a biomarker for atherosclerosis (103). Recent research has further shown that Salusin-β outperforms salusin-α as a biomarker for atherosclerosis (104). Serum Salusin-β levels are significantly associated with the incidence and severity of CAD (105), suggesting that Salusin-β in serum may serve as a potential biomarker for diagnosing and reflecting the progression of CAD. It is noteworthy that coronary artery ectasia (CAE) is a condition closely associated with atherosclerosis, and Salusin-β predicts CAE with a sensitivity of 78.9% and specificity of 75.0% (106). ANRIL and circANRIL are known to be closely linked to endothelial injury and inflammatory responses associated with atherosclerosis (55). Recent studies indicate that a clinical diagnostic model incorporating ANRIL and conventional risk factors has a sensitivity of 84% and specificity of 96%, which can be utilized for the early diagnosis and treatment of CAD (107). FOXO3a is involved in regulating the inflammatory response and apoptosis in endothelial cells by modulating alternative splicing and gene expression (108). Interestingly, studies have identified an association between the risk of CAD and the *rs12196996* polymorphism located in the flanking intron of *circFOXO3* (109).

Milk Fat Globule-EGF Factor 8 (MFGE8) is a protein that encodes epidermal growth factor 8, mediating the inflammatory response in atherosclerosis linked to risk factors such as hypertension and hyperglycemia (110). It plays a critical role in the calcification and senescence of VSMCs induced by high-glucose human umbilical vein endothelial cell exosomes, closely associated with inflammatory responses mediated by IL-1β, IL-6, and IL-8 (111). Another study shows that *MFGE8* participates in the Angiotensin II-mediated inflammatory response associated with the NF-κB signaling pathway, and its knockout reduces levels of MCP-1, TNF-α, ICAM-1, and VCAM-1 produced as a result of NF-κB activation (112). Notably, recent research has identified that the intronic insertion *rs534125149* and splice site variants (splicing receptor variant *rs201988637*) in *MFGE8* are linked to the prevention of coronary artery atherosclerosis (40). Moreover, MFG-E8 levels correlate with the Thrombolysis in Myocardial Infarction (TIMI) risk score (113). The aforementioned RECK plays a role in the inflammatory response in atherosclerosis, and its various splice variants can act as biomarkers for CAD. Long RECK and short RECK exhibit differential expression in patients with stable and unstable angina pectoris, with short RECK being expressed at lower levels in acute myocardial infarction, potentially linked to RECK’s function as an MMP inhibitor and its anti-inflammatory effects (88). Recent research has additionally shown that empagliflozin can inhibit the proliferation and migration of VSMCs by increasing RECK expression (85). Serine-arginine protein kinase (SRPK) modulates the function of Serine/Arginine-rich (SR) proteins through phosphorylation, thereby altering the balance of vascular endothelial growth factor (VEGF) splice variants (114).Research indicates that the SRPK1 inhibitor DBS1 effectively disrupts the binding of SRPK to its substrates (115), resulting in a shift in VEGF splicing from pro-angiogenic to anti-angiogenic forms, highlighting its significant potential in the treatment of cardiovascular diseases. These findings suggest the potential of splice event-related molecules as targets for the diagnosis and treatment of atherosclerosis (**Figure 2**). However, further research on splice events associated with AS inflammatory responses is still lacking. Continued investigation into RNA splicing mechanisms and the development of new intervention strategies will facilitate advancements in AS diagnosis and treatment.

## 6 Perspectives and challenges

The inflammatory response is a key driver of atherosclerosis and will likely become one of the primary approaches for treating cardiovascular diseases in the future. Our study elucidates the molecular mechanisms linking abnormal splice events to the inflammatory response in atherosclerosis, offering a novel perspective on the role of splice events in this condition. Specifically, various splice events mediate endothelial cell injury, macrophage differentiation, and the proliferation and migration of VSMCs in atherosclerosis through the inflammatory response. Their interrelated interactions contribute to the progression of atherosclerosis, further underscoring the significance of splice events in the inflammatory response associated with atherosclerosis. Nonetheless, several unresolved questions persist. While molecules associated with splice events are implicated in the inflammatory response in atherosclerosis, their specific mechanisms of action and the ways in which they influence the functions of other cells remain unclear. Further research is needed to understand how modulating splice events affects other cell types, presenting a significant challenge for future studies. Additionally, we observed a correlation between splice molecules and the risk of atherosclerosis, which supports their potential as diagnostic biomarkers for atherosclerosis. However, future validation through large-scale studies will be necessary. Despite the significant therapeutic potential of targeting splice events in the future, several challenges exist in their clinical application. The effective delivery of splice-targeted therapies remains a primary obstacle, highlighting the need for new carrier systems to improve the delivery and efficiency of these treatments. Splicing factors generally engage in the splicing of multiple genes, meaning that targeting these factors could simultaneously influence various splice events and other biological processes, which raises important considerations regarding treatment specificity. Additionally, prolonged interventions could induce immune responses and impact cellular functions, necessitating further research into the safety of long-term applications. In summary, future research needs to tackle these challenges to ensure the efficacy, specificity, and safety of splice-targeted interventions in atherosclerosis.

## 7 Conclusion

Overall, our research elucidates the molecular mechanisms through which abnormal splicing events mediate inflammation and immune responses in atherosclerosis from multiple perspectives. Furthermore, it investigates the potential of splice factors as diagnostic biomarkers and therapeutic targets for atherosclerosis. offering a fresh outlook that could provide valuable insights for future treatments. While existing clinical and basic research has validated the crucial role of splicing events in the inflammatory response associated with atherosclerosis, the specific mechanisms of action still necessitate additional foundational and clinical studies for confirmation. Conversely, we need to further explore the relationship between splice event-mediated inflammatory responses and other pathological mechanisms in atherosclerosis to provide new therapeutic strategies that can enhance treatment outcomes.
